# Book Notices

**Published:** 1869-07

**Authors:** 


### Book Notices.

A Practical Manual on the Treatment of Club-Foot. By Lewis A. Sayre, M.D., Professor of Orthopœdic Surgery in Bellevue Hospital, Medical College, etc., etc. New York: D. Appleton & Co. 1869.

This is a neatly published duodecimo volume of ninety-one pages, judiciously illustrated with cuts. The Author has had an extensive practical experience in the treatment of the class of deformities about which he writes, and has given the profession in this little volume a very concise practical treatise, such as every intelligent practitioner can understand and apply.

For sale by S. C. Griggs & Co., Chicago.

---

A Treatise on the Function of Digestion; its Disorders, and their Treatment. By F. W. Pavy, M.D., F.R.S., Fellow of the Royal College of Physicians, etc., etc. From the Second London Edition. Philadelphia: Henry C. Lea. 1869; pp. 246. Price $2.

This is a neatly published reprint of the Second Edition of Dr. Pavy's work on Digestion and its Derangements. It presents the reader with a good summary of what is at present known concerning the physiological processes concerned in digestion; the pathological changes these processes are capable of undergoing, and the treatment they require. It is a convenient and practical work for the library of the practitioner. We have not had time to read the work in detail, and consequently cannot criticise any of its details.

---

The Structural Lesions of the Skin: Their Pathology and Treatment. Illustrated. By Howard F. Damon, A.M., M.D., Fellow of the Massachusetts Medical Society; Member of American Medical Association, etc., etc., etc. Pp. 255. Price $3.00. Philadelphia: J. B. Lippincott & Co. 1869.

This is a new work, published in beautiful style, and embraces subjects of practical interest to the profession. The author divides the diseases of which he treats into three classes, namely: Hypertrophies; Atrophies; and Pathological New Formations. We think it will be found a very interesting and profitable work, both for the student and the practitioner.

For sale by S. C. Griggs & Co.

---

Descriptive Catalogue of the Pathological Museum of the Pennsylvania Hospital. By Wm. Pepper, M.D., Physician to the Philadelphia Hospital; Curator and Pathologist to the Pennsylvania Hospital; Lecturer on Morbid Anatomy in the University of Pennsylvania, etc., etc. Philadelphia: Collins, Printer, 1869. Published by the Board of Managers.

This is a well-bound octavo volume, of 138 pages, consisting of a catalogue of the pathological specimens in the Museum of the Hospital, to which is added nineteen pages of description by the author. It is a volume of interest to all students of pathology, and especially to such as expect to visit the Museum.

For sale by W. B. Keen & Cooke, Chicago.

---

Proceedings of the American Pharmaceutical Association at the Sixteenth Annual Meeting, held in Philadelphia, Pa., September, 1868. Also the Constitution and Roll of Members. Philadelphia: Merriher & Sons, Printers, 1869.

This is an octavo volume, of over 500 pages, in paper cover. In addition to the record of proceedings of the Society, it contains a large number of reports and papers on all topics connected with the progress of pharmacy, the purity of drugs, the tariff, and other drug laws, etc., etc. We wish its contents could be read by every Member of Congress as well as by the larger part of the medical profession.

The volume is for sale in this city by E. H. Sargent, druggist. Price $2.50.

